# LPDNet: A Lightweight Network for SAR Ship Detection Based on Multi-Level Laplacian Denoising

**DOI:** 10.3390/s23136084

**Published:** 2023-07-01

**Authors:** Congxia Zhao, Xiongjun Fu, Jian Dong, Cheng Feng, Hao Chang

**Affiliations:** 1 Beijing Institute of Technology, Beijing 100081, China; zcx09890989@163.com (C.Z.); radarvincent@sina.com (J.D.); dafeng7@126.com (C.F.); 3120200660@bit.edu.cn (H.C.); 2 Tangshan Research Institute of BIT, Tangshan 063000, China

**Keywords:** synthetic aperture radar (SAR), Convolutional Neural Network (CNN), target detection, trainable, Laplacian pyramid denoising

## Abstract

Intelligent ship detection based on synthetic aperture radar (SAR) is vital in maritime situational awareness. Deep learning methods have great advantages in SAR ship detection. However, the methods do not strike a balance between lightweight and accuracy. In this article, we propose an end-to-end lightweight SAR target detection algorithm, multi-level Laplacian pyramid denoising network (LPDNet). Firstly, an intelligent denoising method based on the multi-level Laplacian transform is proposed. Through Convolutional Neural Network (CNN)-based threshold suppression, the denoising becomes adaptive to every SAR image via back-propagation and makes the denoising processing supervised. Secondly, channel modeling is proposed to combine the spatial domain and frequency domain information. Multi-dimensional information enhances the detection effect. Thirdly, the Convolutional Block Attention Module (CBAM) is introduced into the feature fusion module of the basic framework (Yolox-tiny) so that different weights are given to each pixel of the feature map to highlight the effective features. Experiments on SSDD and AIR SARShip-1.0 demonstrate that the proposed method achieves 97.14% AP with a speed of 24.68FPS and 92.19% AP with a speed of 23.42FPS, respectively, with only 5.1 M parameters, which verifies the accuracy, efficiency, and lightweight of the proposed method.

## 1. Introduction

Synthetic aperture radar (SAR) is an active microwave sensor with strong penetrability, which produces high-resolution images at all-time and all-weather. It is widely used in disaster prevention, emergency rescue, land resources monitoring, urban management, etc. [1,2]. Ship detection based on SAR images effectively monitors ocean conditions and manages ocean resources [3].

Traditional SAR ship detection methods are mainly based on the statistical features [4,5,6], scattering characteristics [7], and transform domain [8], while the most typical method is the constant false alarm rate (CFAR) [9,10,11,12]. However, its feature extraction process is complex, and it is sensitive to speckle noise and complex backgrounds, which can not meet the requirements of high-precision, lightweight, and real-time SAR ship detection.

With the emergence and vigorous development of CNNs, object detection algorithms based on deep learning have become the mainstream methods of image processing. The algorithms fall into two main categories: one-stage [13,14,15,16] and two-stage [17,18,19,20] methods. The one-stage algorithms, including Yolo [13], SSD [14], RetinaNet [15], etc., produce the detection results directly from the figures. The two-stage algorithms, such as Faster R-CNN [18], Mask R-CNN [19], and Cascade R-CNN [20], divide the target detection into two stages, generating region proposal and classification. In recent years, with the proposal of SAR datasets, containing SSDD [21], HRSID [22], and OpenSARship [23], SAR target detection based on deep learning has developed towards high precision, high speed, and lightweight. The two-stage methods generally possess higher accuracy. Y. Li et al. [24] designed a feature relay amplification and multiscale feature jump connection structure. They proposed a lightweight network based on Faster R-CNN [18] to improve the detection speed while ensuring detection accuracy. Cui et al. [25] densely connected CBAM [26] to each concatenated feature map from top to bottom of the pyramid network to obtain abundant features containing resolution and semantic information and improve the detection accuracy of multiscale ships. The one-stage detectors reduce the amount of calculation through various methods to improve detection efficiency. H. Wan et al. [27] designed a lightweight backbone network based on Yolox [28], highlighting SAR targets’ unique strong scattering characteristics by integrating channels and spatial attention mechanisms and improving detection accuracy and speed. Qi et al. [29] introduced weak segmentation and attention mechanism [30] into a single-stage detector to obtain richer semantic information. Mao et al. [31] proposed a simple detector based on U-Net [32], but the detection performance was unsatisfactory. These methods are competitive in terms of accuracy and lightweight. However, they process SAR images as optical images and ignore their differences. SAR images are produced in microwave/millimeter wave bands, which are different from common three-channel images, RGB or HSV, acquired by visible and partial infrared band sensors. The differences make the CNN-based methods not fully applicable to SAR image processing. Moreover, because of the basic principle of coherent imaging, SAR images contain a lot of speckle noise, which brings a low signal-to-noise ratio.

In SAR images, speckle noise can easily lead to false detection and target missing. Traditional image denoising mainly includes spatial domain-based methods and frequency domain-based methods. L. Liu et al. [33] used morphological filtering for SAR image denoising to preprocess image change detection. R. Farhadiani et al. [34] used a maximum a posteriori (MAP) estimator to denoise in the complex wavelet domain and utilized the local pixel group filtering based on the non-local principal component analysis (LPG-PCA) method to smooth the homogeneous areas and enhance the details. Through feature extraction of CNNs, SAR image denoising methods based on deep learning extract the effective features and obtain the noise and details in the image. J. Zhang et al. [35] performed SAR image denoising by a multiconnection network that incorporates wavelet features while maintaining the texture structure of the image. S. Liu et al. [36] extracted shallow features from noisy images by designing kernels of different sizes to form multiscale modules. Following this, the shallow features are mapped to the residual dense dual-attention network to obtain the deep features of SAR images, and the final denoised images are generated through global residual learning. However, most of the above methods only regard SAR image denoising as the preprocessing and do not realize the end-to-end image processing. At the same time, in the traditional denoising methods, the noise suppression thresholds are often set according to experience, which is vulnerable to subjective influence, and the self-adaptation to each image, each network, and target detection task cannot be achieved.

This article proposes an integrated model of the transform domain denoising method and target detection network, LPDNet, to solve the above problems. The main contributions can be summarized as follows:

(1) To reduce speckle noise and improve detection performance, this article proposes a novel intelligent denoising method based on Laplacian pyramid denoising and CNN. Through CNN-based threshold suppression, the multi-level Laplacian denoising becomes adaptive to every SAR image via back-propagation based on the subsequent detection network, which makes the denoising processing supervised.
(2) This article proposes to perform channel modeling on feature maps composed of spatial sub-bands, denoised maps, and original maps. It strengthens the balance between the contributions of spatial and frequency features, targets, and noise, thereby effectively combining the multi-dimensional information.
(3) In order to further improve the accuracy of the model, the CBAM is introduced to the feature fusion module of the basic framework. The CBAM is flexible and lightweight, which avoids a lot of computational overhead, and the accuracy is compensated.
(4) In this article, supervised thinking in deep learning is combined with traditional image decomposition and enhancement methods. It provides a new supervised denoising method for SAR target detection. The method can be embedded in SAR image processing as a preprocessing process.

The feasibility and effectiveness of LPDNet are verified on the SAR ship detection data set (SSDD), AIR SARShip-1.0 [37], and HRSID. The method is compared with other CNN-based algorithms and evaluated by indicators, such as average precision mean (mAP), precision, recall, F1-score, frame per second (FPS), and parameters. The experimental results prove its accuracy and efficiency.

The remainder of this article is organized as follows: Section 2 introduces our proposed target detection method, Section 3 presents experiments and results analysis, and Section 4 concludes the paper.

## 2. Methodology

In this section, the multi-level Laplacian pyramid denoising network is developed, and details of the implementation procedures are presented. First, an overview of LPDNet is introduced. Then, the structure of multi-level Laplacian pyramid denoising is developed. Finally, CBAM is introduced into the model to enhance the representation ability.

### 2.1. Processing Flow of LPDNet

**Figure 1:**
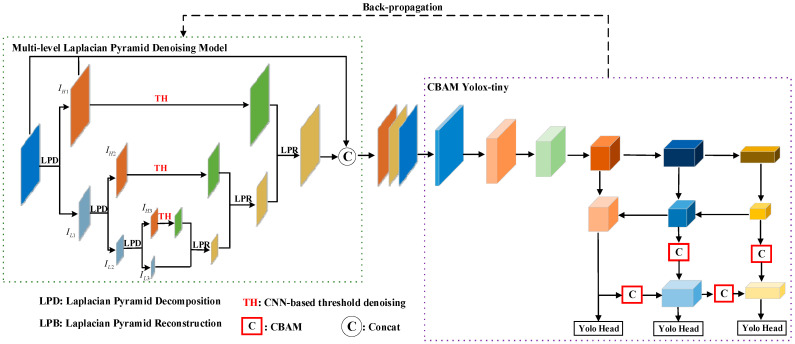
Processing flow of the proposed network.

The architecture of the proposed network, LPDNet, is shown in Figure 1. LPDNet firstly derives the Laplacian pyramid intelligent denoising module to reduce speckle noise. The multi-level Laplacian pyramid transformer is applied to decompose the image into different frequency sub-bands. A module based on CNN is used to intelligently determine the threshold of each high-frequency sub-band through back-propagation. The process of back-propagation in Figure 1 is depicted as a dotted arrow. The adaptive thresholds are generated and extracted through the forward propagation and adjusted through the back-propagation so that the thresholds can be adaptively determined by the detection task. Then, hard thresholding is used to suppress the noise. The denoised image can be obtained through Laplacian pyramid reconstruction. To compensate for the feature lost in the denoising and enhance the high-frequency information, channel modeling is performed. The three-channel image consists of a high-frequency image of the first level of the Laplacian pyramid decomposition, a denoised image, and an original image. After that, CBAM is introduced into the feature fusion part of the Yolox-tiny to enhance feature extraction. The details of the method are shown as follows.

### 2.2. Multi-Level Laplacian Pyramid Denoising Model

Laplacian pyramid transform is a multiscale and multiresolution image processing method that can decompose an image’s high-frequency and low-frequency features into sub-bands of different resolutions according to different scales. Speckle noise in SAR images mainly distributes in the high-frequency sub-bands of the image, with relatively small coefficients. By threshold suppression, the coefficients that are lower than the threshold can be compressed. Then, the denoised image can be obtained by Laplacian pyramid reconstruction. However, the traditional threshold determination method is usually affected by subjective factors, which cannot achieve the self-adaptation of each image, each network, and target detection task. In this section, a CNN-based Laplacian pyramid denoising method is proposed, which converts the threshold into a trainable variable through the network. The denoising threshold of each high-frequency sub-band can be determined intelligently through the supervised learning of the subsequent detection network.

#### 2.2.1. Laplacian Pyramid Denoising Model

**Figure 2:**
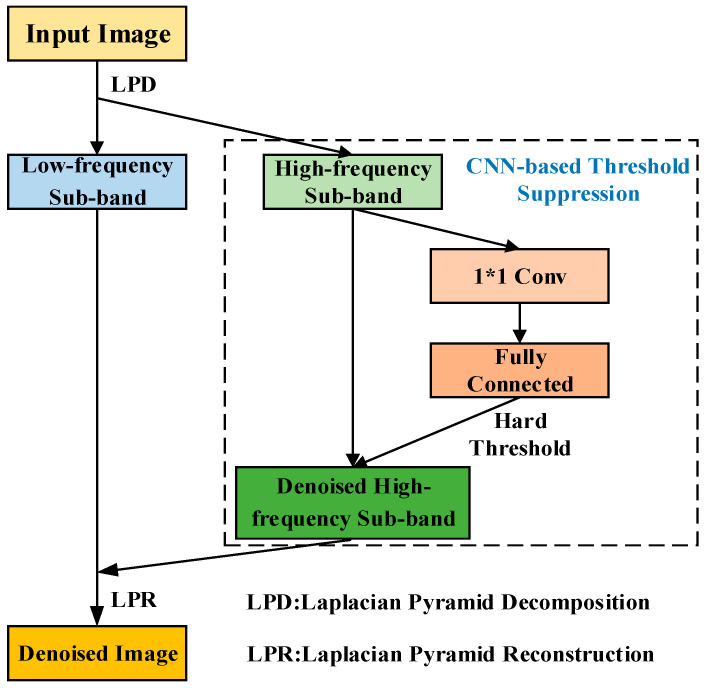
Laplacian pyramid denoising model.

The Laplacian pyramid denoising model is shown in Figure 2, where LPD is the Laplacian pyramid decomposition, and LPR is the Laplacian pyramid reconstruction. The high-frequency and low-frequency sub-bands are obtained by Laplacian pyramid decomposition, and the high-frequency sub-band is put into the threshold suppression module for noise suppression. The CNN-based threshold suppression module consists of a convolution layer, a fully connected layer, and a hard threshold function. The convolution kernel size of the convolution layer is the same as the size of the input high-frequency sub-band image and has the global receptive field of the image, which can be expressed as:

(1) $$O_{i}=MaxPool(f_{i}*I_{H}+b_{i})$$

where $O_{i}$ is the *i*th feature channel of the convolutional layer output, MaxPool() is the maximum pooling function, $f_{i}$ represents the convolution kernel of the input feature corresponding to the *i*th channel, $I_{H}$ is the high-frequency sub-band image input to the convolution layer, and $b_{i}$ represents the biasing of the convolutional layer. The fully connected layer takes the output of the convolutional layer, $O_{i}$, as the input. The output (D) is a tensor, $D\in R^{1\times 1\times 1}$:

(2) D=ReLU(WO+b)

where ReLU() is the relu activation function, which can be defined as follows:

(3) ReLU(x)={x,x>00,x≤0

Then, the denoising threshold corresponding to a high-frequency sub-band can be obtained. The noise in the high-frequency sub-band is suppressed by a hard threshold function as follows:

(4) $${\hat{I}}_{H}(x,y)=hard\_threshold(I_{H}(x,y))=\{\begin{array}{c} 0.001\times I_{H}(x,y),I_{H}(x,y)\leq D\\ I_{H}(x,y),I_{H}(x,y)>D \end{array}$$

where $I_{H}(x,y)$ and ${\hat{I}}_{H}(x,y)$ represent the high-frequency sub-band coefficients before and after the threshold suppression, respectively.

Finally, the Laplacian pyramid reconstruction is encapsulated into an image reconstruction layer. The low-frequency sub-band image and the denoised high-frequency sub-band image are put into the layer to obtain the denoised image.

The Laplacian denoising module based on the CNN can be embedded in any image processing network as the preprocessing. The convolution layer and the fully connected layer can be trained by the loss function through the back-propagation. The denoising threshold can be calculated by the global characteristics of each image to achieve intelligent denoising.

#### 2.2.2. Multi-Level Laplacian Pyramid Denoising Model

**Figure 3:**
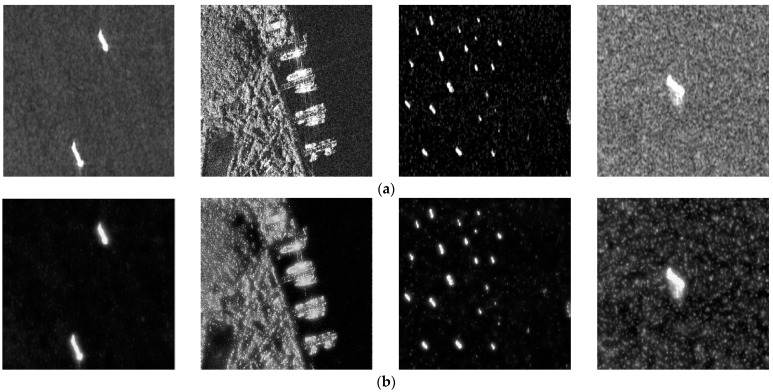
Original images and denoised images in the SSDD; (**a**) original images, (**b**) denoised images.

A multi-level Laplacian pyramid denoising model is designed based on the single-level denoising structure proposed in Section 2.2.1. The model contains three layers, as shown in the green box in Figure 1. Firstly, the high-frequency sub-band $I_{H1}$ and low-frequency sub-band $I_{L1}$ are obtained through the first layer of Laplacian pyramid decomposition. By decomposing the first and second low-frequency sub-bands, $I_{H2}$, $I_{L2}$ and $I_{H3}$, $I_{L3}$ can be obtained, respectively. Secondly, the CNN-based threshold suppression module is constructed for each level’s high-frequency sub-bands to obtain the sub-bands after noise suppression. Then, the denoised images are obtained by Laplacian pyramid reconstruction, as shown in Figure 3. The images in the first row are the originals in the SSDD, and the ones in the second row are the corresponding denoised images. It is obvious that the speckle noise in the denoised images is weaker than that in the original images. However, the hard threshold function may cause image distortion, including the ringing effect and pseudo-Gibbs phenomenon.

#### 2.2.3. CBAM Yolox-Tiny

The Yolox networks, which are more accurate and faster than other detection algorithms, are derived from Yolo v3. Considering the accuracy, speed, and lightweight requirements of ship detection, this article uses Yolox-tiny as the basic network.

**Figure 4:**
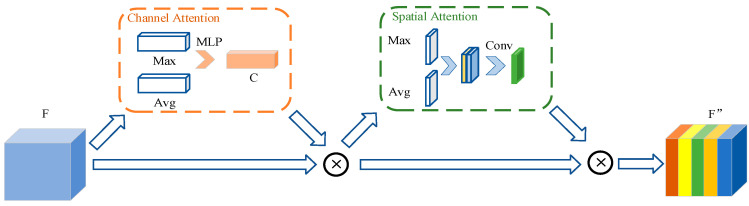
Structure of CBAM.

CBAM proposed by Woo et al. [35] contains channel and spatial attention, which deduces the attention maps along the channel and spatial dimension, as shown in Figure 4. Channel attention is obtained by utilizing the relations of the features between channels. Specifically, the global information is obtained by two kinds of pooling: maximum pooling and average pooling. The nonlinear characteristic change is performed through a fully connected network. Then, the attention weight is obtained by summing and activating the two channels. The process can be expressed as follows:

(5) $$M_{c}(F)=\sigma (MLP(AvgPool(F))+MLP(MaxPool(F)))$$

where *F* is the original feature map, σ is the sigmoid function, and *MLP* indicates multi-layer perceptron. Similarly, two maps can be obtained by using maximum pooling and average pooling on the channel dimensions. The spatial attention weight can be obtained by concatenating two maps and the convolution operation.

(6) $$M_{s}(F)=\sigma (f^{7\times 7}([AvgPool(F);MaxPool(F)]))$$

Here, $f^{7\times 7}$ represents the 7 × 7 convolution process.

By multiplying the attention maps with the feature image and adapting the features, the essential features are focused, and unnecessary features are suppressed, so that the representation ability is enhanced.

**Figure 5:**
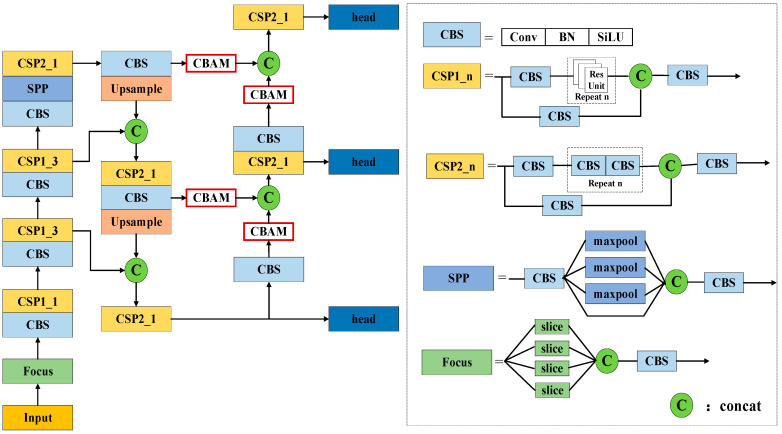
CBAM Yolox-tiny structure.

CBAM is introduced into the feature fusion layer of the Yolox-tiny network, as shown in Figure 5. It is placed on the two branches of the network. Different weights are given to each channel and pixel of the feature map, which provides adequate information for feature extraction. At the same time, the invalid information is suppressed, thus the detection accuracy is further improved.

## 3. Experiments

In this section, the experiments are discussed and analyzed to verify the effect of the proposed method through experiments on the SSDD, AIR SARShip-1.0 and HRSID. The experiments discuss the effectiveness of each part of the network and the comparative effect with other networks. All models are implemented using Pytorch framework under Linux system with NVIDIA Tesla K80 GPUs support.

### 3.1. Dataset

To verify the model’s effectiveness, experiments are carried out on SSDD, AIR SARShip-1.0, and HRSID. The SSDD dataset contains 1160 SAR images, each with a size of about 500 × 500. These images have resolutions of 1–15 m, with different polarization modes, including HH, HV, VH, and VV. There are 2358 ships with different scales and materials. The AIR SARShip-1.0 contains 31 images of large scenes with a size of 3000 × 3000 and nearly a thousand ships of different types, with image resolutions of 1 m and 3 m and a single polarization mode. The HRSID includes 5604 images and 16,951 ships with different polarization modes and a ranging resolution from 1 m to 5 m. In the experiment, we randomly resize the images into 416 × 416, 416 × 416, and 800 × 800, respectively. All datasets contain sea surfaces with different sea conditions and complex scenes, including inshore and offshore. For the convenience of the experiment, the data sets are divided into a train set, a test set, and a validation set according to the ratio of 7:2:1.

### 3.2. Evaluation Metrics

In the experiments, precision, recall, average precision (*AP*), and *F1* are employed to evaluate the performance of the methods. Precision and recall can be described as follows:

(7) $$Precision=\frac{TP}{TP+FP}$$

(8) $$Recall=\frac{TP}{TP+FN}$$

where *TP* means the ships are detected correctly, *FP* means missed detection, and *FN* means false alarm.

*AP* and *F1* measure the balance between precision and recall:

(9) $$AP=\int_{0}^{1}P(R)\cdot RdR$$

(10) $$F1=\frac{2\cdot R\cdot P}{R+P}$$

where *P* respects precision and *R* respects recall, *AP* computes the average value of precision over the interval from recall = 0 to 1, and *F1* is the harmonic mean of precision and recall. Both of them are comprehensive calculation indicators of precision and recall, and the higher the value, the better the detection effect.

### 3.3. Evaluation of the Methods with Different Thresholds

In this section, the effect of the intelligent Laplacian denoising in the LPDNet is compared with the methods based on fixed thresholds to verify the effectiveness of the CNN-based threshold determination method.

**Figure 6:**
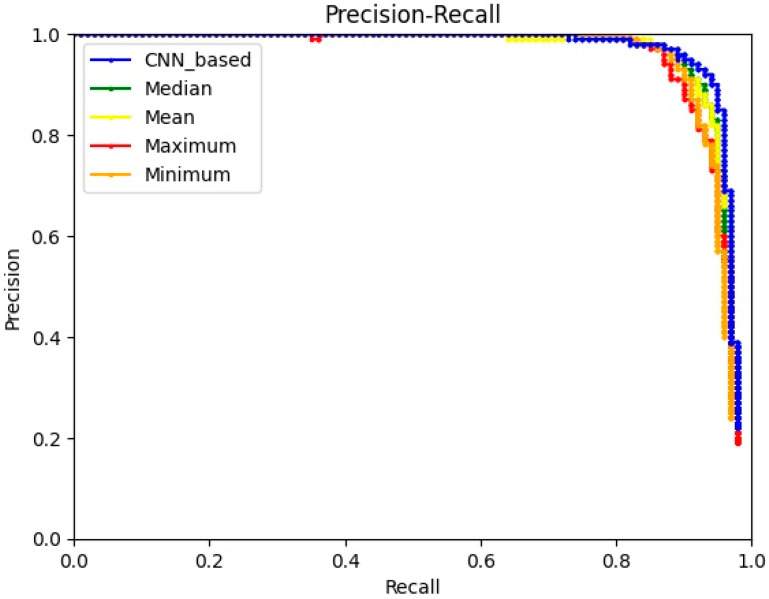
PR curves of methods with different thresholds.

From the network combining Yolox-tiny with the multi-level Laplacian pyramid denoising model (LP Yolox-tiny), the value ranges of the three intelligently determined thresholds are [0, 1.143], [0, 0.579], and [0, 0.881], respectively. We take the medians, means, maximums, and minimums of the ranges as the thresholds of the experiments, respectively. The rest of the module settings are the same as the LP Yolox-tiny. As shown in Table 1, the effects of methods with fixed thresholds are inferior to that of the CNN-based method (LP Yolox-tiny). In particular, the AP of LP Yolox-tiny achieves 96.25%, which is 0.51%, 0.5%, 1.43%, and 1.28% higher than that of the methods taking medians, means, maximums, and minimums as thresholds, respectively. Compared with the fixed threshold, the intelligently determined threshold makes the denoising effect more suitable for the detection network, so the detection effect is advantageous. The advantages of the CNN-based method can be further reflected by the precision-recall (PR) curve, as shown in Figure 6.

### 3.4. Ablation Experiment

In this section, the effect of the Laplacian denoising and CBAM in the LPDNet are evaluated based on the SSDD from visual and indicator aspects.

#### 3.4.1. Analysis of Visualization Effects

**Figure 7:**
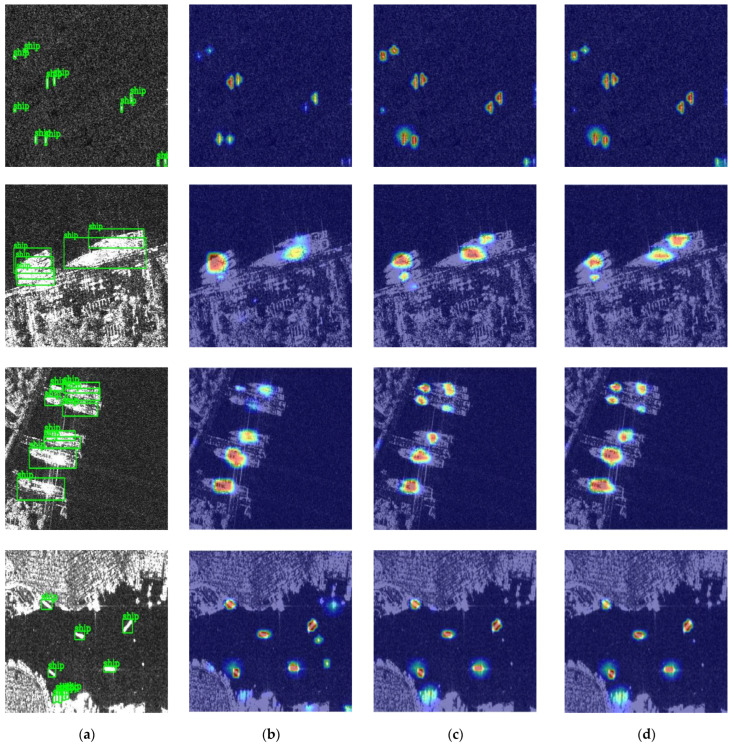
Some visualization results. (**a**) Ground truth; (**b**) Yolox-tiny; (**c**) LP Yolox-tiny; (**d**) LPDNet.

To observe the feature extraction effect of the network, heatmaps are adopted to visualize the effect of the network. As shown in Figure 7, the Laplacian denoising model and the CBAM can reduce speckle noise and enhance high-frequency information and effective features. The modules improve the detection effect, especially for the small, dense, parallel, and inshore targets. In the first row of Figure 7, the Laplacian denoising model effectively highlights the small targets in the image with speckle noise through denoising and improves the feature extraction effect of small targets. In the second and third rows, the parallel ships in the complex background are separated through the network. The locations of the ships are predicted more accurately. Moreover, as shown in the fourth row, the LPDNet extracts the features of the densely arranged inshore ships and pays more attention to them. At the same time, the false alarm rate of small, suspected targets in the figure is reduced.

#### 3.4.2. Analysis of Detection Results

**Figure 8:**
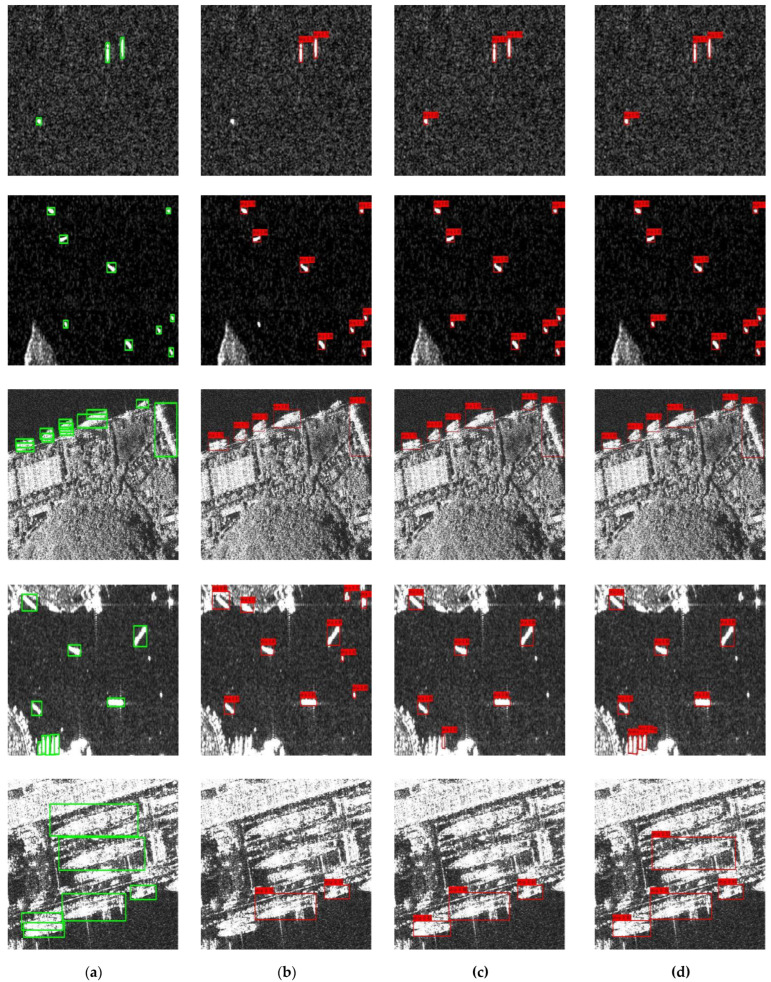
The results of ablation experiments. (**a**) Ground truth; (**b**) Yolox-tiny; (**c**) LP Yolox-tiny; (**d**) LPDNet.

As shown in Figure 8, the images contain different scenes and resolutions. The multi-level Laplacian pyramid denoising model can remove the speckle noise with large amplitude, highlight the small targets, and reduce missed detection. In the first and second rows of Figure 8, LP Yolox-tiny improves the accuracy of small target detection in the case of high noise. The denoising part is helpful in detecting small targets in noisy scenes as the confusing noise is depressed. At the same time, high-frequency information in the three-channel image provides more boundary information that can improve the effect of target detection. As shown in the third row, high-frequency contour features are beneficial to the extraction of target and coast features, which improve the detection effect of inshore targets. The CBAM can adaptively refine features, focus on important features, and suppress unnecessary features by deducing the attention map along the channel and space dimensions to increase the representation ability. As shown in the fourth and fifth rows, the CBAM makes the network pay more attention to the target features. The detection of densely arranged inshore ships and parked ships in the port is more accurate. The detection results in Table 2 further demonstrate the effectiveness of each module.

### 3.5. Comparison Experiment

In this section, the comparison experiments are carried out on the SSDD and AIR SARShip-1.0, including SSD, CenterNet, Yolo v4-tiny, Yolo v7, and Yolox-tiny. Since the two datasets contain images of various scenes, polarization modes, and resolutions, the experimental results are robust and universal. The comparison experiments are carried out on the same train and test datasets, and the default parameters of the models are used.

**Figure 9:**
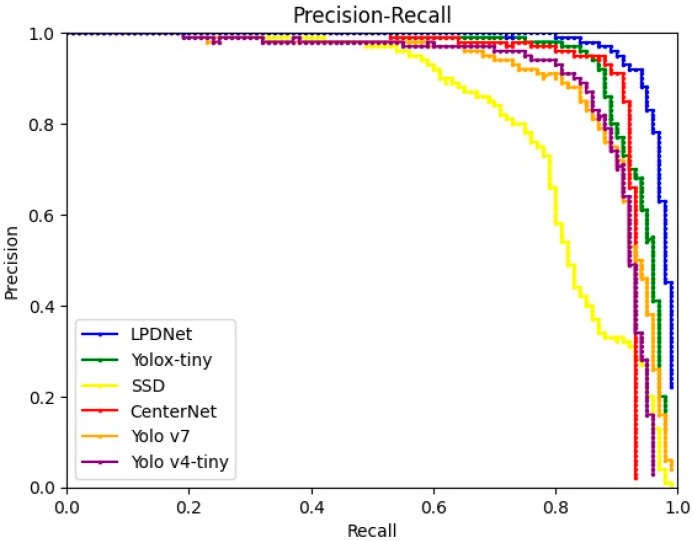
Precision-recall (PR) curves of different algorithms on the SSDD.

**Figure 10:**
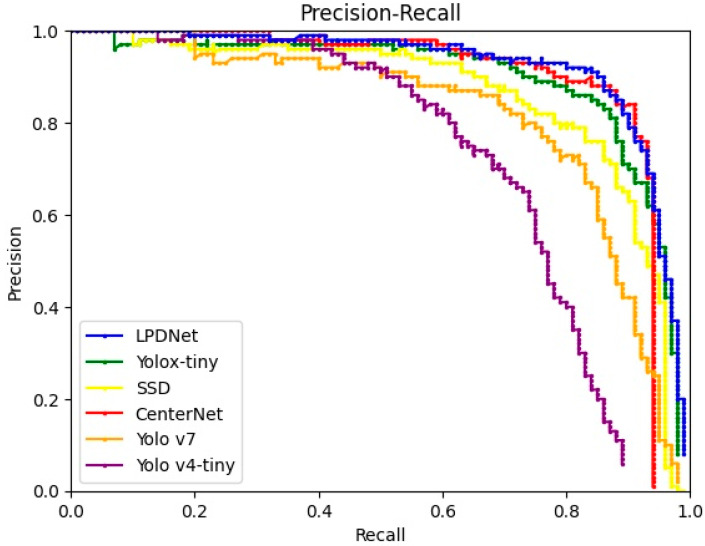
Precision-recall (PR) curves of different algorithms on the AIR SARShip-1.0.

The results of the experiments are shown in Table 3 and Table 4. On the SSDD, the AP of the LPDNet achieves 97.14%, which is 15.13%, 5.91%, 6.92%, 7.34%, and 3.46% higher than the SSD, CenterNet, Yolo v7, Yolo v4-tiny, and Yolox-tiny, respectively. The parameters of SSD, CenterNet, and Yolo v7 are several times the proposed algorithm, while the lightweight methods, Yolo v4-tiny and Yolox-tiny, have similar numbers of parameters as the proposed algorithm. Meanwhile, lightweight algorithms take less time to test than the algorithm proposed, but the accuracies of the algorithms are inferior. On the AIR SARShip-1.0 dataset, the comparison algorithms have similar effects. The methods’ precision-recall (PR) curves on the SSDD and AIR SARShip-1.0 are shown in Figure 9 and Figure 10, respectively. The results of the LPDNet are excellent. On the one hand, the intelligent denoising module adaptively denoises each image to provide a clear image for the feature extraction module. At the same time, high-frequency images provide more explicit boundary information for the feature extraction process and strengthen the feature extraction effect. On the other hand, CBAM highlights the effective features, suppresses the invalid features, and enhances the target detection effect. The LPDNet improves both the precision and recall considerably, reaching the best performance in the mentioned algorithms, while considering both lightweight and detection speed. To sum up, the algorithm in this article further improves the accuracy of target detection on the premise of ensuring lightweight, which indicates a better detection effect.

### 3.6. Migration Ability

To test the migration ability of LPDNet, we performed an experiment on the HRSID. We divided the entire dataset according to the dataset division of the original paper. Table 5 shows the detection results on the HRSID. The proposed method can achieve state-of-the-art results, with AP, F1, recall, and precision values of 93.09%, 0.89, 85.99%, and 93%, respectively. The results show that the proposed method has a strong migration ability.

## 4. Conclusions

In this article, a new lightweight SAR ship detector, LPDNet, is proposed. Based on the multiresolution characteristics of Laplacian pyramid transformation, a CNN-based method is used to obtain the denoising threshold from the global characteristics of each image in a supervised way to achieve intelligent denoising. At the same time, channel modeling is adopted to make up for the loss of information during denoising, enhance the contour features, and improve the detection effect. Moreover, CBAM is introduced into the feature fusion module to enhance the ability of feature representation and further improve the network performance. Experiments show that the proposed network achieves a balance between lightweight and detection accuracy and has an excellent detection effect.

In the future, the generalization performance of LPDNet will be studied, including validation on large scene datasets, and the effects of image resolution, polarization mode, and other conditions on target detection. We will lighten the detector without sacrificing accuracy and obtain better detection results. Further, we will explore the application of the multi-level Laplacian pyramid denoising model in target recognition, segmentation, and other fields. In addition, more intelligent denoising methods will be explored. We will focus on combining traditional denoising methods and CNNs so that the traditional denoising methods can develop intelligence and embeddability to serve in image interpretation.

## Figures and Tables

**Table 1:** The results of experiments with different thresholds.

Methods	Threshold1	Threshold2	Threshold3	Recall	Precision	AP	F1
**Median**	0.024	0	0.056	89.01%	96.05%	95.74%	0.92
**Mean**	0.087	0.024	0.083	89.01%	94.55%	95.75%	0.92
**Maximum**	1.143	0.579	0.881	87.55%	94.09%	94.82%	0.91
**Minimum**	0	0	0	89.01%	94.37%	94.97%	0.92
**CNN-based**	--	--	--	91.03%	94.67%	96.25%	0.93

**Table 2:** The results of ablation experiments.

Methods	Recall	Precision	AP	F1	Params	FPS
**Yolox-tiny**	86.81%	92.94%	93.68%	0.9	5,032,866	30.94
**LP Yolox-tiny**	91.03%	94.67%	96.25%	0.93	5,144,200	25.19
**LPDNet (proposed)**	90.66%	95.74%	97.14%	0.93	5,150,158	24.68

**Table 3:** The results of different methods on SSDD.

Methods	Recall	Precision	AP	F1	Params	FPS
**SSD**	56.41%	95.36%	82.01%	0.71	23,612,246	8.161
**CenterNet**	72.98%	97.49%	91.23%	0.83	32,664,197	18.93
**Yolo v7**	77.29%	90.75%	90.22%	0.83	37,194,710	14.88
**Yolo v4-tiny**	83.86%	89.76%	89.8%	0.87	5,874,116	54.05
**Yolox-tiny**	86.81%	92.94%	93.68%	0.9	5,032,866	30.94
**LPDNet (proposed)**	90.66%	95.74%	97.14%	0.93	5,150,158	24.68

**Table 4:** The results of different methods on AIR SARShip-1.0.

Methods	Recall	Precision	AP	F1	Params	FPS
**SSD**	76.73%	82.2%	86.11%	0.79	23,612,246	7.71
**CenterNet**	67.07%	94.02%	90%	0.78	32,664,197	14.17
**Yolo v7**	68.64%	84.06%	81.61%	0.76	37,194,710	14.65
**Yolo v4-tiny**	61.77%	77.7%	72.98%	0.69	5,874,116	47.85
**Yolox-tiny**	86.15%	83.83%	89.96%	0.85	5,032,866	29.94
**LPDNet (proposed)**	88.64%	84.88%	92.19%	0.87	5,150,158	26.76

**Table 5:** The results of different methods on HRSID.

Method	Recall	Precision	AP	F1
Faster R-CNN	77.5%	88.8%	78.2%	0.83
Cascade R-CNN	79.3%	89.9%	79.2%	0.84
SSD	85.3%	87.4%	88.8%	0.86
CenterNet	87.4%	81.8%	86.3%	0.85
RetinaNet	83.8%	69.8%	82.5%	0.76
FCOS	79.5%	91.9%	86.6%	0.85
Yolox-tiny	82.33%	94.02%	91.08%	0.88
LPDNet (proposed)	85.99%	93%	93.09%	0.89

## Data Availability

Not applicable.
